# Do Changes in Hemodynamic Parameters Depend Upon Length of Sleep Deprivation? Comparison Between Subjects With Normal Blood Pressure, Prehypertension, and Hypertension

**DOI:** 10.3389/fphys.2018.01374

**Published:** 2018-09-28

**Authors:** Joanna Słomko, Monika Zawadka-Kunikowska, Sławomir Kujawski, Jacek J. Klawe, Małgorzata Tafil-Klawe, Julia L. Newton, Paweł Zalewski

**Affiliations:** ^1^Department of Hygiene, Epidemiology and Ergonomics, Faculty of Health Sciences, Nicolaus Copernicus University in Toruń, Toruń, Poland; ^2^Department of Human Physiology, Faculty of Medicine, Nicolaus Copernicus University in Toruń, Toruń, Poland; ^3^Institute for Cellular Medicine, The Medical School, Newcastle University, Newcastle upon Tyne, United Kingdom

**Keywords:** hypertension, cardiovascular, autonomic nervous system, sleep deprivation, blood pressure

## Abstract

The main objective of the study was to analyze the impact of sleep deprivation upon hemodynamic and autonomic parameters in subjects with normal blood pressure (BP) compared to prehypertension and hypertension at 24, 28, and 32 h of total sleep deprivation (TSD). Thirty volunteers, healthy men with current medical tests indicating the absence of disease took part in the study. After physical examination (basic neurological, clinical examination, echocardiography and doppler ultrasound of the renal arteries, evaluation of the autonomic nervous system) subjects were divided into three groups: I – normotensive, II – pre-hypertensive, III – hypertensive (age: 31.2 ± 2.1 vs. 33.5 ± 2.7 vs. 36.8 ± 2.7 years, *p* > 0.05; BMI: 25.2 ± 0.8 vs. 29.0 ± 1.5 vs. 26.4 ± 1.0 kg/m^2^, *p* > 0.05). Hemodynamic and autonomic parameters were automatically measured at rest and in a tilted position with a Task Force Monitor. The Task Force Monitor consists of electrocardiography, impedance cardiography, oscillometric, and continuous BP measurement. Mixed models with random effects was applied in order to analyze the parameters’ dependence on the time and the group of patients. One-way ANOVA or Kruskal–Wallis test were used to detect differences between normotensive, pre-hypertensive and hypertensive groups in each time point. In the pre-hypertensive group 28-h TSD resulted in increased vagal outflow [changes in high frequency heart rate (HR) variability, *p* = 0.0189], as evidenced by decreased HR (*p* = 0.0293). Moreover after 24-h TSD and 28-h TSD we observed changes in BP parameters. In hypertensive group, the most important changes in hemodynamic parameters: systolic blood pressure (sBP, *p* = 0.0031), diastolic blood pressure (dBP, *p* = 0.0136), cardiac output (CO, *p* = 0.0439) and changes in HR (*p* = 0.0063) after tilt test were observed after 32-h TSD. In conclusion, our results show that changes in hemodynamic parameters during sleep deprivation depend on the baseline BP and duration of TSD. What is important, both groups reported a decrease of sBP and dBP during the TSD (pre-hypertensive group after 24, 28-h TSD; hypertensive group after 32-h TSD. In our opinion, this is the first study which considers three homogenous groups in terms of gender: only men, during different points of acute TSD: 24, 28, and 32 h of TSD in laboratory condition.

## Introduction

Sleep deprivation (SD) is a growing health problem and is related with changes in lifestyle behaviors and an increased prevalence of sleep disorders (e.g., insomnia, obstructive sleep apnea) and neurological disorders. Epidemiological studies suggest a link between short sleep duration and an increased incidence of cardiovascular diseases occurring via the activation of various pathways: neural autonomic control changes, altered inflammatory response or increased oxidative stress (Tobaldini et al., 2013b, 2014, 2017). Current literature on the impact of SD upon the function of the autonomic nervous system are not conclusive. Holmes et al. (2002) indicate that 30 h of SD lowers activity of sympathetic autonomic control manifested by a decrease in heart rate (HR), with no change in parasympathetic autonomic nervous system activity. Other authors have observed that after 24-h SD, HR and blood pressure (BP) were higher compared to baseline in healthy subjects. Zhong et al. (2005) claimed that 36 h of SD increases activity of sympathetic autonomic nervous system function, with a reduction in activity of parasympathetic function part and a decrease in baroreflex sensitivity. A few studies found no changes in the autonomic profile induced by chronic SD (Muenter et al., 2000). SD studies of hypertensive subjects have shown significant increases in BP and sympathetic nervous system function after SD (Lusardi et al., 1999). What’s important, compared to acute deprivation, chronic SD studies are more difficult to perform and results of these studies are not homogeneous; the studies differ in terms of study group characteristics, experimental settings, and experimental timetables.

The main objective of the study was to analyze the impact of total sleep deprivation (TSD) upon hemodynamic and autonomic parameters in subjects with normal BP, prehypertension, and hypertension at different periods of TSD. In our opinion, this is the first study which applies three homogenous groups in terms of gender: only men, during different point of acute TSD: 24, 28, and 32 h of TSD in laboratory condition.

## Materials and Methods

### Study Group

**FIGURE 1:**
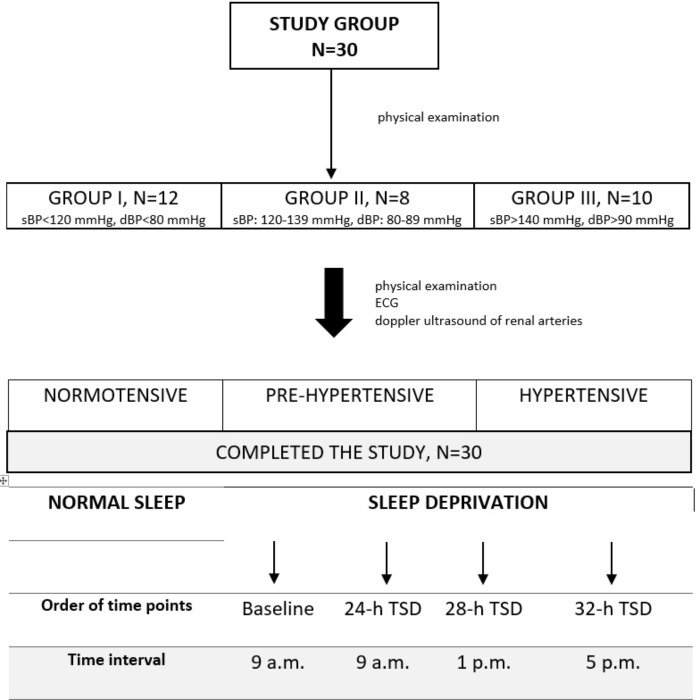
Study protocol.

Thirty volunteers, healthy men with current medical tests indicating the absence of disease (including routine laboratory tests) took part in the study. In addition to giving their voluntary consent to participation in the study, the main enrollment criteria included sex: male, no co-morbidity, no reported sleep disorders (Pittsburgh Sleep Quality Index < 5; Buysse et al., 1989), no extreme chronotype, [ratings between 14 and 21 points on the morning-evening M/E questionnaire (Torsvall and Akerstedt, 1980)]. Exclusion criteria consisted of factors that could possibly modify the response to SD: shift work, caffeine, alcohol, drugs dependence, participation in sports at competitive level, alcohol consumption within 12 h before the test, receiving any medication/supplements during the study and potential disorders of the cardiovascular system observed during the test. All potential study participants were questioned about their sleep quality, life habits and health state. Pre-test health state assessment of subjects included: basic neurological, clinical examination, evaluation of the autonomic nervous system using the Autonomic Symptom Profile (Suarez et al., 1999). After physical examination subjects were divided into three groups: I – normotensive, II – pre-hypertensive, III – hypertensive (age: 31.2 ± 2.1 years vs. 33.5 ± 2.7 years vs. 36.8 ± 2.7 years, *p* > 0.05; BMI: 25.2 ± 0.8 kg/m^2^ vs. 29.0 ± 1.5 kg/m^2^ vs. 26.4 ± 1.0 kg/m^2^, *p* > 0.05) (**Figure 1**). Prehypertension and primary hypertension was found based on a physical examination, the collected data included symptoms that may indicate the secondary hypertension, the presence of risk factors, comorbidities, family interviews of hypertension and cardiovascular and renal diseases, and the objective one, i.e., the two measurements of BP in the sitting position during two different visits, echocardiography and doppler ultrasound of the renal arteries. The study was approved by the Ethics Committee, Ludwik Rydygier Memorial Collegium Medicum in Bydgoszcz, Nicolaus Copernicus University, Toruń; written informed consent was obtained from all of the participants.

### Total Sleep Deprivation (TSD) Protocol

One week prior to the study (baseline week), the participants were requested to abstain from excessive caffeine (one caffeine-containing beverage per day at most) and alcohol consumption (<5 alcoholic beverages per week). Subjects arrived at the laboratory at 08:30 a.m. after their typical sleep at home the night before (total sleep time, TST = 432.6 ± 61.2 min). TSD began at 9 a.m. and finished at 5 p.m. after 32 h of continuous wakefulness. The examination was performed in the chronobiology laboratory while maintaining constant conditions (constant routine, temperature 22°C, humidity 60%, light < 10 lx). Light intensity control is crucial due to the known differences in HR variability (HRV) between dim and bright light conditions during sleep deprivation. Subjects ate the same meals at the same time of the day (8:00, 12:00, 15:00, 19:30). Water (100 ml) was administered at hourly intervals during the protocol. Reading, writing, talking, and playing games were allowed during the experiment. To make sure that patients did not fall asleep they were cared for by trained personnel. During the TSD, two investigators were present in the laboratory, rotations of 12 h were organized. Additionally, the device Actigraph GT3X (Actigraph, Pensacola, FL, United States) was used during the experiment to monitor subjects’ sleep deprivation (Santos-Lozano et al., 2013).

### Cardiovascular Measurements

Hemodynamic [HR, BP, stroke volume (SV), cardiac output (CO), total peripheral resistance (TPR)] and autonomic parameters [low frequency (LF), high frequency (HF)] were automatically measured at rest (15 min after stabilization of the signals) and in a tilted position (10 min after stabilization of the signals) with a Task Force Monitor – TFM (CNS Systems, Gratz, Austria). Task Force Monitor^®^ is designed for non-invasive measurements of hemodynamic parameters and consist of electrocardiography (ECG), impedance cardiography (ICG), oscillometric (oscBP), and continuous (contBP) blood pressure measurement (Fortin et al., 1998, 2006; Buszko et al., 2017).

Measurements of cardiovascular system parameters were obtained at 4 points during the study: 9 a.m. (baseline) on the first day of the study, 9 a.m. (24-h TSD), 1 p.m. (28-h TSD), 5 p.m. (32-h TSD) of the second day.

### Statistical Analyses

Mixed models with random effects based on two-way ANOVA was applied in order to analyze the parameters’ dependence on the time and the group of patients. The Maximum likelihood method was applied for estimating variance parameters. The analyses were performed with R version 3.5.0 (R: library lme) and Matlab 2017b.

One-way ANOVA or Kruskal–Wallis test were used to detect differences between groups considering the group effect on cardiovascular parameters and HRV. If significant differences were observed by ANOVA, *post hoc* pair-wise comparisons were performed using Tukey’s test. When significant differences were found in Kruskal–Wallis test, a multiple comparison of rank sum test was performed. The level of significance for all tests was set at *p* < 0.05. All calculations were conducted with STATISTICA 13.0 PL statistical package (StatSoft).

## Results

**FIGURE 2:**
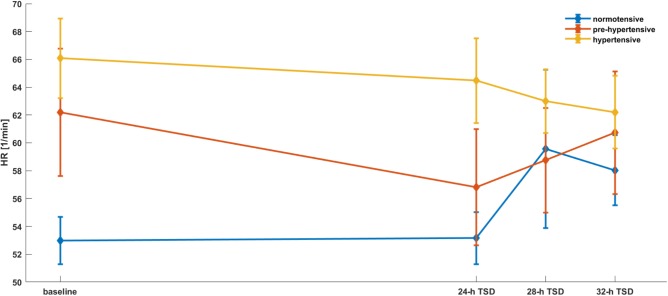
HR parameters’ dependence on the time and the group of patients. Mixed models with random effects based on two-way ANOVA indicate that there was a significant effect of time (*p* = 0.0238), group (*p* = 0.0087) and both group × time (II group after 28-h TSD; III group after 28 and 32-h TSD). Interestingly, both groups reported a decrease of HR during the TSD compared to baseline.

**FIGURE 3:**
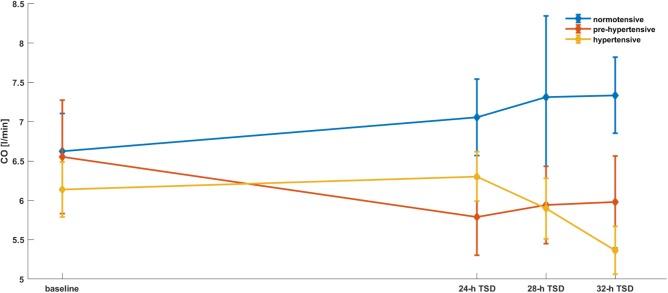
CO parameters’ dependence on the time and the group of patients. Mixed models with random effects based on two-way ANOVA indicate that there was a significant effect of time × group (*p* = 0.0439). After 32-h TSD CO was significantly lower in the III group compared to baseline.

**FIGURE 4:**
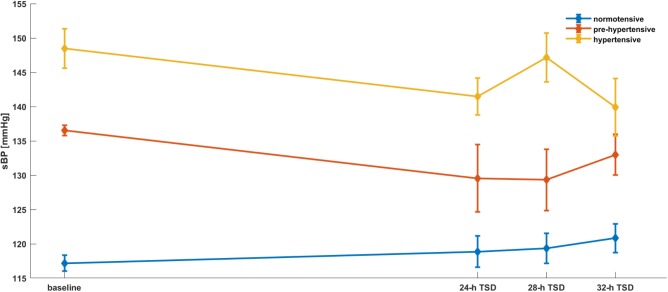
sBP parameters’ dependence on the time and the group of patients. Mixed models with random effects based on two-way ANOVA indicate that there was a significant effect of group (*p* = 0.0001) and both group × time (II group after 24, 28-h TSD; III group after 32-h TSD). What is important, both groups reported a decrease of sBP during the TSD compared to baseline.

**FIGURE 5:**
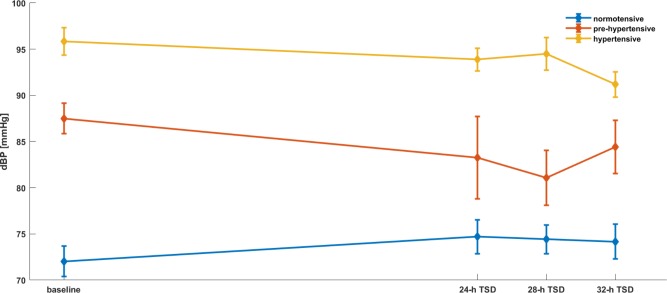
dBP parameters’ dependence on the time and the group of patients. Mixed models with random effects based on two-way ANOVA indicate that there was a significant effect of group (*p* = 0.0001) and both group × time (II group after 24, 28-h TSD; III group after 32-h TSD). Interestingly, both groups reported a decrease of dBP during the TSD compared to baseline.

To assess the effect of time and the groups of patients in discussed parameters we applied mixed models with random effects. The estimation of the model for hemodynamic and spectral parameters were presented in the **Tables 1**–**3** and **Figures 2**–**5**. A significant changes were found after 28-h TSD in HR at the pre-hypertensive and hypertensive groups. 24-h and 28-h TSD influence BP parameter – significant changes in sBP and dBP were observed after 24-h and 28-h TSD in the pre-hypertensive and after 24-h (only sBP) and 32-h TSD in hypertensive group. CO changes were observed in hypertensive group, after 32-h TSD. Only 28-h TSD influence autonomic parameters – that changes were observed in HF-RRI in pre-hypertensive group.

**Table 1:** Hemodynamic parameters’ dependence on the time and the group of patients.

Parameter	Variable	Value	SE	*p*-value
HR	Intercept	52.9	3.1	**0.0001**
	Group II vs. group I	9.2	4.9	ns
	Group III vs. group I	13.1	4.6	**0.0087**
	24-h TSD vs. baseline	0.2	2.8	ns
	28-h TSD vs. baseline	6.6	2.8	**0.0238**
	32-h TSD vs. baseline	5.1	2.8	ns
	Group II: 24-h TSD	−5.5	4.5	ns
	Group III: 24-h TSD	−1.8	4.2	ns
	Group II: 28-h TSD	−10.0	4.5	**0.0293**
	Group III: 28-h TSD	−9.6	4.2	**0.0252**
	Group II: 32-h TSD	−6.5	4.5	ns
	Group III: 32-h TSD	−8.9	4.2	**0.0378**
sBP	Intercept	117.2	2.7	**0.0001**
	Group II vs. group I	19.3	4.2	**0.0001**
	Group III vs. group I	31.3	3.9	**0.0001**
	24-h TSD vs. baseline	1.7	2.7	ns
	28-h TSD vs. baseline	2.2	2.7	ns
	32-h TSD vs. baseline	3.6	2.7	ns
	Group II: 24-h TSD	−8.7	4.3	**0.0453**
	Group III: 24-h TSD	−8.7	4.0	**0.0329**
	Group II: 28-h TSD	−9.4	4.3	**0.0304**
	Group III: 28-h TSD	−3.5	4.0	ns
	Group II: 32-h TSD	−7.2	4.3	ns
	Group III: 32-h TSD	−12.2	4.0	**0.0031**
dBP	Intercept	72.0	1.9	**0.0001**
	Group II vs. group I	15.5	2.9	**0.0001**
	Group III vs. group I	23.8	2.9	**0.0001**
	24-h TSD vs. baseline	2.7	1.8	**0.0001**
	28-h TSD vs. baseline	2.4	1.8	ns
	32-h TSD vs. baseline	2.1	1.8	ns
	Group II: 24-h TSD	−6.9	2.9	**0.0188**
	Group III: 24-h TSD	−4.6	2.7	ns
	Group II: 28-h TSD	−8.8	2.9	**0.003**
	Group III: 28-h TSD	−3.7	2.7	ns
	Group II: 32-h TSD	−5.2	2.9	ns
	Group III: 32-h TSD	−6.7	2.7	**0.0136**
SV	Intercept	124.9	7.1	**0.0001**
	Group II vs. group I	−16.1	11.1	ns
	Group III vs. group I	−31.5	10.5	**0.0056**
	24-h TSD vs. baseline	7.5	4.1	ns
	28-h TSD vs. baseline	−4.1	4.1	ns
	32-h TSD vs. baseline	1.6	4.1	ns
	Group II: 24-h TSD	−10.6	6.5	ns
	Group III: 24-h TSD	−2.8	6.1	ns
	Group II: 28-h TSD	0.1	6.4	ns
	Group III: 28-h TSD	4.3	6.0	ns
	Group II: 32-h TSD	−8.0	6.5	ns
	Group III: 32-h TSD	−8.3	6.0	ns
CO	Intercept	6.2	0.5	**0.0001**
	Group II vs. group I	−0.1	0.8	ns
	Group III vs. group I	−0.5	0.8	ns
	24-h TSD vs. baseline	0.4	0.5	ns
	28-h TSD vs. baseline	0.7	0.5	ns
	32-h TSD vs. baseline	0.7	0.5	ns
	Group II: 24-h TSD	−1.2	0.8	ns
	Group III: 24-h TSD	−0.3	0.7	ns
	Group II: 28-h TSD	−1.3	0.8	ns
	Group III: 28-h TSD	−0.9	0.7	ns
	Group II: 32-h TSD	−1.3	0.8	ns
	Group III: 32-h TSD	−1.7	0.5	**0.0439**
TPR	Intercept	1113.7	97.1	**0.0001**
	Group II vs. group I	262.7	153.5	ns
	Group III vs. group I	432.8	144.0	**0.0057**
	24-h TSD vs. baseline	−40.8	71.6	ns
	28-h TSD vs. baseline	37.6	71.6	ns
	32-h TSD vs. baseline	−77.9	71.6	ns
	Group II: 24-h TSD	118.9	113.3	ns
	Group III: 24-h TSD	−54.8	106.2	ns
	Group II: 28-h TSD	−43.6	113.3	ns
	Group III: 28-h TSD	11.9	106.2	ns
	Group II: 32-h TSD	139.9	113.3	ns
	Group III: 32-h TSD	194.2	106.2	ns

*Group I – normotensive, group II – pre-hypertensive, group III – hypertensive. TSD, total sleep deprivation. Bolded values = values which are statistically significant, *p* < 0.05.*

**Table 2:** Spectral parameters’ dependence on the time and the group of patients.

Parameter	Variable	Value	SE	*p*-value
LF-RRI	Intercept	1391.0	332.6	**0.0001**
	Group II vs. group I	311.6	525.9	ns
	Group III vs. group I	−642.8	493.3	ns
	24-h TSD vs. baseline	−76.5	360.3	ns
	28-h TSD vs. baseline	399.1	360.3	ns
	32-h TSD vs. baseline	−387.2	360.3	ns
	Group II: 24-h TSD	−547.1	569.7	ns
	Group III: 24-h TSD	126.8	534.4	ns
	Group II: 28-h TSD	−760.2	569.7	ns
	Group III: 28-h TSD	−142.1	534.4	ns
	Group II: 32-h TSD	−51.4	569.7	ns
	Group III: 32-h TSD	570.1	534.4	ns
HF-RRI	Intercept	1189.4	583.6	**0.0448**
	Group II vs. group I	290.7	922.8	ns
	Group III vs. group I	−752.4	865.7	ns
	24-h TSD vs. baseline	−77.8	656.5	ns
	28-h TSD vs. baseline	1572.4	656.5	**0.0189**
	32-h TSD vs. baseline	−150.1	656.5	ns
	Group II: 24-h TSD	−228.3	1037.9	ns
	Group III: 24-h TSD	239.4	973.7	ns
	Group II: 28-h TSD	−1987.4	1037.9	**0.0591**
	Group III: 28-h TSD	−1401.3	973.7	ns
	Group II: 32-h TSD	161.1	1037.9	ns
	Group III: 32-h TSD	509.9	973.7	ns

*Bolded values = values which are statistically significant, *p* < 0.05.*

**Table 3:** Magnitude of changes (delta) induced by tilt test on hemodynamic parameters’ dependence on the time and the group of patients.

Parameter	Variable	Value	SE	*p*-value
Δ HR	Intercept	17.1	4.0	**0.0001**
	Group II vs. group I	−6.4	6.4	ns
	Group III vs. group I	−12.1	6.0	**0.0525**
	24-h TSD vs. baseline	−0.8	3.9	ns
	28-h TSD vs. baseline	−2.7	3.9	ns
	32-h TSD vs. baseline	40.9	3.9	**0.0001**
	Group II: 24-h TSD	−0.2	6.2	ns
	Group III: 24-h TSD	−1.0	5.8	ns
	Group II: 28-h TSD	4.4	6.2	ns
	Group III: 28-h TSD	3.5	5.8	ns
	Group II: 32-h TSD	9.1	6.2	ns
	Group III: 32-h TSD	16.3	5.8	**0.0063**
Δ sBP	Intercept	18.7	8.2	**0.0249**
	Group II vs. group I	−8.3	12.9	ns
	Group III vs. group I	−19.7	12.2	ns
	24-h TSD vs. baseline	3.2	2.9	ns
	28-h TSD vs. baseline	2.7	2.9	ns
	32-h TSD vs. baseline	3.8	2.9	ns
	Group II: 24-h TSD	6.4	4.6	ns
	Group III: 24-h TSD	−0.2	4.3	ns
	Group II: 28-h TSD	1.9	4.6	ns
	Group III: 28-h TSD	−4.2	4.3	ns
	Group II: 32-h TSD	0.7	4.6	ns
	Group III: 32-h TSD	−0.4	4.3	ns
Δ dBP	Intercept	25.9	6.5	**0.0002**
	Group II vs. group I	−10.9	10.3	ns
	Group III vs. group I	−18.6	9.7	ns
	24-h TSD vs. baseline	3.1	2.6	ns
	28-h TSD vs. baseline	2.8	2.5	ns
	32-h TSD vs. baseline	3.6	2.5	ns
	Group II: 24-h TSD	6.3	4.0	ns
	Group III: 24-h TSD	0.7	3.8	ns
	Group II: 28-h TSD	1.7	4.0	ns
	Group III: 28-h TSD	−2.1	3.8	ns
	Group II: 32-h TSD	0.2	4.0	ns
	Group III: 32-h TSD	−1.5	3.8	ns

*Bolded values = values which are statistically significant, p < 0.05.*

Differences between normotensive, pre-hypertensive and hypertensive groups in each time point were presented in the **Tables 4**–**6**. *Post hoc* analysis revealed significant differences in baseline between normotensive and hypertensive in HR, sBP, dBP, SV, TPR, HF-RRI, between normotensive and pre-hypertensive in sBP, dBP; 24-h SD between normotensive and hypertensive in HR, sBP, dBP, SV, TPR; 28-h SD between normotensive and hypertensive in sBP, dBP, mBP, HF-RRI and between pre-hypertensive and hypertensive in sBP, dBP; 32-h SD between normotensive and hypertensive in sBP, dBP, SV, TPR and between normotensive and pre-hypertensive in dBP and TPR.

**Table 4:** Hemodynamic parameters at baseline and after 24-h, 28-h, 32-h SD in normotensive, pre-hypertensive, and hypertensive groups in resting conditions.

Parameter	Time	Group I mean ± SD	Group II mean ± SD	Group III mean ± SD	I vs. II *p*-value	I vs. III *p*-value	II vs. III *p*-value
HR	**Baseline**	52.9 ± 6.0	62.2 ± 13.0	66.1 ± 9.0	ns	**0.0101**	ns
	**24-h TSD**	53.2 ± 6.5	56.8 ± 11.8	64.5 ± 9.7	ns	**0.0262**	ns
	**28-h TSD**	59.5 ± 19.7	58.7 ± 10.6	63.0 ± 7.2	ns	ns	ns
	**32-h TSD**	58 ± 8.7	60.7 ± 12.4	62.2 ± 8.2	ns	ns	ns
sBP	**Baseline**	116.2 ± 4.0	137.5 ± 2.1	148.5 ± 9.2	**0.0352**	**0.0001**	ns
	**24-h TSD**	118.9 ± 7.8	129.6 ± 14.0	141.5 ± 8.4	ns	**0.0004**	ns
	**28-h TSD**	119.3 ± 7.6	129.3 ± 12.6	147.2 ± 11.2	ns	**0.0001**	**0.0059**
	**32-h TSD**	120.8 ± 7.2	133.0 ± 8.4	140.0 ± 13.2	ns	**0.0006**	ns
dBP	**Baseline**	72.0 ± 5.7	87.5 ± 4.7	95.8 ± 4.6	**0.0168**	**0.0001**	ns
	**24-h TSD**	74.7 ± 6.3	83.3 ± 12.6	93.9 ± 3.9	ns	**0.0002**	ns
	**28-h TSD**	74.4 ± 5.4	81.1 ± 8.4	94.5 ± 5.5	ns	**0.0001**	**0.0009**
	**32-h TSD**	74.2 ± 6.5	84.4 ± 8.1	91.2 ± 4.3	**0.0091**	**0.0001**	ns
SV	**Baseline**	124.9 ± 25.0	108.9 ± 35.4	93.5 ± 15.5	ns	**0.0258**	ns
	**24-h TSD**	132.5 ± 25.1	105.8 ± 30.9	98.2 ± 11.4	ns	**0.0036**	ns
	**28-h TSD**	120.8 ± 26.0	104.9 ± 32.3	93.7 ± 16.5	ns	ns	ns
	**32-h TSD**	126.5 ± 20.8	102.5 ± 34.3	86.7 ± 13.3	ns	**0.002**	ns
CO	**Baseline**	6.6 ± 1.6	6.6 ± 2.0	6.1 ± 1.1	ns	ns	ns
	**24-h TSD**	7.1 ± 1.7	5.8 ± 1.4	6.3 ± 1.0	ns	ns	ns
	**28-h TSD**	7.3 ± 3.6	5.9 ± 1.4	5.9 ± 1.2	ns	ns	ns
	**32-h TSD**	7.3 ± 1.7	6.0 ± 1.7	5.4 ± 1.0	ns	**0.015**	ns
TPR	**Baseline**	1113.7 ± 315.6	1376.4 ± 388.6	1546.5 ± 340.8	ns	**0.0317**	ns
	**24-h TSD**	1072.9 ± 302.5	1454.6 ± 435.7	1450.8 ± 251.9	ns	**0.0374**	ns
	**28-h TSD**	1151.3 ± 378.7	1370.4 ± 295.9	1596.1 ± 407.3	ns	ns	ns
	**32-h TSD**	1035.7 ± 277.6	1438.4 ± 381.3	1662.8 ± 250.0	**0.0319**	**0.0003**	ns

*Bolded values = values which are statistically significant, *p* < 0.05.*

**Table 5:** Spectral parameters at baseline and after 24-h, 28-h, 32-h SD in normotensive, pre-hypertensive and hypertensive groups in resting conditions.

Parameter	Time	Group I mean ± SD	Group II mean ± SD	Group III mean ± SD	I vs. II *p*-value	I vs. III *p*-value	II vs. III *p*-value
LF-RRI	**Baseline**	1391.1 ± 838.5	1702.6 ± 2404.5	748.2 ± 846.5	ns	ns	ns
	**24-h TSD**	1314.6 ± 1208.0	1079.0 ± 840.0	798.6 ± 791.0	ns	ns	ns
	**28-h TSD**	1790.2 ± 1300.1	1341.6 ± 1178.7	1005.3 ± 1032.9	ns	ns	ns
	**32-h TSD**	1003.8 ± 810.0	1264.0 ± 1036.8	931.1 ± 1023.5	ns	ns	ns
HF-RRI	**Baseline**	1189.4 ± 786.9	1480.1 ± 2975.1	436.9 ± 514.5	ns	**0.0418**	ns
	**24-h TSD**	1111.5 ± 1148.7	1173.9 ± 1922.9	598.5 ± 560.8	ns	ns	ns
	**28-h TSD**	2761.8 ± 4638.3	1065.1 ± 1634.4	608.1 ± 643.9	ns	**0.0183**	ns
	**32-h TSD**	1039.3 ± 1016.8	1491.1 ± 266.6	796.9 ± 872.9	ns	ns	ns

*Bolded values = values which are statistically significant, *p* < 0.05.*

**Table 6:** Magnitude of changes (delta) induced by tilt test on hemodynamic parameters at baseline and after 24-h, 28-h, 32-h SD in normotensive, pre-hypertensive, and hypertensive groups.

Parameter	Time	Group I mean ± SD	Group II mean ± SD	Group III mean ± SD	I vs. II *p*-value	I vs. III *p*-value	II vs. III *p*-value
Δ HR	**Baseline**	17.1 ± 6.1	10.6 ± 4.0	11.7 ± 7.9	ns	ns	ns
	**24-h TSD**	16.3 ± 5.7	9.6 ± 5.0	11.2 ± 8.9	ns	ns	ns
	**28-h TSD**	18.7 ± 7.0	12.3 ± 6.8	13.0 ± 7.5	ns	ns	ns
	**32-h TSD**	16.6 ± 7.8	9.8 ± 3.9	14.0 ± 8.0	ns	ns	ns
Δ sBP	**Baseline**	18.7 ± 4.1	10.4 ± 9.4	14.4 ± 11.3	ns	ns	ns
	**24-h TSD**	22.0 ± 9.0	20.0 ± 8.4	16.2 ± 9.4	ns	ns	ns
	**28-h TSD**	21.4 ± 6.5	15.0 ± 5.0	13.6 ± 7.3	ns	**0.0457**	ns
	**32-h TSD**	22.5 ± 4.7	14.9 ± 5.6	16.7 ± 6.6	**0.0266**	ns	ns
Δ dBP	**Baseline**	26.0 ± 5.3	15.0 ± 6.1	19.3 ± 4.4	**0.0011**	**0.0421**	ns
	**24-h TSD**	29.0 ± 9.1	24.4 ± 9.6	22.6 ± 10.1	ns	ns	ns
	**28-h TSD**	28.8 ± 6.3	19.3 ± 4.2	20.7 ± 5.7	**0.0022**	**0.032**	ns
	**32-h TSD**	29.6 ± 5.7	18.9 ± 6.7	20.7 ± 4.2	**0.006**	**0.0094**	ns

*Bolded values = values which are statistically significant, p < 0.05.*

## Discussion

The major finding of the present study is the evidence that variability of hemodynamic parameters depends upon sleep deprivation timetables – the most important changes in pre-hypertensive group were after 28-h TSD and in hypertensive group after 32-h TSD. First, in the pre-hypertensive group 28-h TSD resulted in increased vagal outflow (manifested in HF-RRI changes), as evidenced by decreased HR. Moreover after 24-h TSD and 28-h TSD we observed changes in BP parameters. Second, in the hypertensive group the most important changes in hemodynamic parameters: BP parameters, CO and changes in HR after tilt test were observed after 32-h TSD. Third, after 28-h TSD BP parameter differences between groups were observed not only between normotensive and hypertensive but also between the pre-hypertensive and hypertensive group. Moreover in the pre-hypertensive and hypertensive group we observed significant changes in tilt test: they had lower sBP increase after tilt in comparison to normotensive group. The observed changes may indicate less-effective regulatory mechanisms in these groups. Thus, we might speculate that chronic sleep deprivation might slowly lead to a pathological condition.

There are only a limited number of studies on the effect of sleep deprivation on autonomic function in subjects with primary/essential hypertension. The main response of the cardiovascular and autonomic nervous system to TSD in hypertensive patients is increased sympathetic nervous activity during the night and the following morning, leading to increased BP and HR (Lusardi et al., 1999; Klawe et al., 2002; Tafil-Klawe et al., 2007). More often in the literature is described the influence of stress factors on the homeostasis of the human body. Sleep deprivation, sleep fragmentation or shift work are an important stress factor that is associated with disruption of the natural circadian rhythm. Esler et al. (1991) noticed that higher rates of norepinephrine spillover into the cerebrovascular circulation after experimental laboratory stressor were in patients with essential hypertension than in healthy subjects, suggesting an underlying increase in central nervous system norepinephrine turnover may be the basis for the increased sympathetic outflow. In primary hypertension (particularly in younger patients), a differentiated activation of the sympathetic outflow to the heart and kidneys is present, based on measurements of norepinephrine spillover to plasma (Esler et al., 1991). Moreover, other studies show that during the mental stress test, the BP response was significantly greater in subjects with positive than with negative family histories (Widgren et al., 1992).

Despite numerous studies, the causes and mechanisms of hypertension have not been fully understood. Authors suggest that the central nervous system mechanisms underlying this disease and the channels which allow these mechanisms are funneled to the peripheral autonomic nervous system and trigger this cardiovascular disorder.

Several pathologies including hypertension, diabetes and obstructive sleep apnea have been associated with changes in carotid bodies activity (Conde et al., 2017). Increase in carotid sinus nerve activity drives excitation in medullary presympathetic pathways and is integrated in the brain stem to induce cardiorespiratory compensatory responses. Augmented chemoreceptor tonic drive and hypoxic ventilatory response were observed in young men with arterial hypertension. In the young normotensive subjects with a family background of hypertension and in the young spontaneously hypertensive rats, an increased sensitivity to chemoreceptor stimulation is seen before the onset of hypertension (Trzebski et al., 1982; Tafil-Klawe et al., 1985, Klawe et al., 1991). These observations suggest that peripheral chemoreceptor overactivity plays a causal role in the development of hypertension. Increase in BP after sleep deprivation, increase in sympathetic activity following increased tonic drive from arterial chemoreceptor, in young men with normal baroreceptor activity activate arterial baroreceptor, causing reflex response – increase in vagal activity. In older patients with hypertension a decrease in baroreceptor sensitivity (resetting of baroreflex) is observed. One possible mechanism is adaptation of the baroreflex to prolonged stimulation (peripheral or central). Our pre-hypertensive and hypertensive subjects were younger, so the cardiovascular response to sleep deprivation might be considered as the baroreceptor’s prevention to increase BP – vagal activity.

The following physiological events, regulating sympathetic activity, should be considered in pre-hypertension and hypertension: augmented peripheral chemoreceptor drive, attenuation of hypoxic chemosensitivity with age, decrease in baroreceptor reactivity in hypertension and in the course of biological aging, interaction between baro- and chemoreflexes (decrease in baroreceptor sensitivity in hypoxic conditions). The process of adaptation of these receptor’s sensitivity, different stages of development of hypertension and family background of hypertension show a mosaic of different probable cardiovascular responses to physiological stimuli.

Our results suggest an increase in parasympathetic dominance after TSD, which may be related to the modulation regulatory and compensatory mechanisms underlying the pathomechanism of the primary hypertension.

The current literature suggesting changes in cardiovascular and autonomic functioning during sleep deprivation is ambiguous. This is most likely because of the experimental timetables (length of SD, hours of SD per day, and total protocol duration), study population characteristics or experimental setting (laboratory conditions vs. real life models) different – often opposite reactions – are observed. Sleep deprivation does not have a commonly accepted definition including the minimum sleep length, it is not considered a disorder, therefore in the available literature the authors use a different value: most often 24–60 h. Several experimental protocols have been carried out to assess the changes induced SD in healthy subjects. One night of sleep deprivation affects cardiovascular autonomic response causing an increase of cardiac sympathetic modulation and reduce the amplitude of cardiovascular autonomic response to gravitational stimulus. Moreover authors observed that SD modifies the immune pro-inflammatory profile, increasing the plasmatic levels of IFN-γ (Tobaldini et al., 2013a). The opposite results were obtained by Vaara et al. (2009) which suggest that 60-h TSD resulted in increased vagal outflow, as evidenced by decreased HR.

In SD studies, selection of participants to the study group seems to be significant, where the differences resulting from gender seem to be the most important. For this reason, the results of our research included a homogeneous group in terms of gender, only men. Findings from the Whitehall II cohort showed gender-specific associations between sleep deprivation and hypertension. Short duration of sleep (<5 h) was associated with higher risks of hypertension only among women (Cappuccio et al., 2007). Carter et al. (2012) suggest that mechanisms underlying the acute hypertensive response to SD differ in men and women and that neural (downward shift of the sympathetic baroreflex operating point in men) and non-neural (reduced testosterone in men) mechanisms may be underlying these sex differences. Experimental settings of SD studies also have a significant influence on the study result: study carried out in shift work (night work conditions) often associated with the occurrence of environmental stress, or in experimental (laboratory) conditions. Pagani et al. (2009) suggest that one night sleep deprivation without stress or disturbances, does not lead to increased arterial pressure values or to changes in autonomic or baroreflex profiles that could conceivably favor hypertension development. Moreover they suggest that previous observations of increased arterial pressure variability levels after one night SD might rather reflect the disturbing effect of psychological stress on autonomic arousal (Pagani et al., 2009).

The results of our research taking place in laboratory show that one night sleep deprivation is too short a time to induce significant changes in hemodynamic parameters in hypertensive patients. However, in pre-hypertensive the BP changes were significant after 24-h TSD. Also, the choice of the test evaluating of the autonomic nervous system function seems to be important. The tilt test used in this experiment is characterized by a diversified response depending on the sex of the subjects – women tended to have lower tilt-table tolerance associated with a smaller splanchnic vasoconstrictor reserve than men (Jarvis et al., 2010). Comparison of the effects of orthostatic stimulation after sleep deprivation are rarely performed. Tobaldini et al. (2013a) suggest that increase of cardiac sympathetic modulation and the reduction of cardiac vagal control induced by tilt test were significantly lowered after 26-h sleep deprivation and this suggests that one night of SD impairs the capability of the physicians to correctly respond to stressor stimuli.

In summary, based on the published evidence, acute SD is able to modify hemodynamic control and autonomic cardiovascular regulation in pre-hypertensive and hypertensive patients.

One limitation of this study was the relatively small number of participants. Most of the available SD studies were conducted on small groups, which is related to the specificity of sleep research and their high cost. The lack of an objective sleep measure, such as polysomnographic recording before the experiment (on the baseline week) and laboratory measurements (such as the aldosterone/renin ratio) must be considered as a limitation of the study. Moreover primary aldosteronism was not included in the screening of secondary hypertension.

However, the strength of the present study is twofold: first, in our opinion this is the first study which compares three homogenous groups: normotensive, pre-hypertensive and hypertensive during different time points of sleep deprivation. Secondly, the results of this study confirm that variability of hemodynamic and autonomic parameters depends upon baseline value of BP and sleep deprivation timetables. Future research is needed in standardized conditions with large-scale studies to clarify the detrimental effects of chronic SD in normotensive and hypertensive patients.

## Author Contributions

JS and PZ contributed conception and design of the study and wrote the first draft of the manuscript. JS, MZ-K, and SK organized the database. MZ-K performed the statistical analysis. JK, MT-K, and JN wrote the sections of the manuscript. All authors contributed to manuscript revision, read and approved the submitted version.

## Conflict of Interest Statement

The authors declare that the research was conducted in the absence of any commercial or financial relationships that could be construed as a potential conflict of interest.
